# Structure of the human cation–chloride cotransporter NKCC1 determined by single-particle electron cryo-microscopy

**DOI:** 10.1038/s41467-020-14790-3

**Published:** 2020-02-21

**Authors:** Xiaoyong Yang, Qinzhe Wang, Erhu Cao

**Affiliations:** Department of Biochemistry, University of Utah School of Medicine, Salt Lake City, UT 84112-5650 USA

**Keywords:** Biochemistry, Structural biology, Electron microscopy, Cryoelectron microscopy

## Abstract

The secondary active cation–chloride cotransporters (CCCs) utilize the existing Na^+^ and/or K^+^ gradients to move Cl^−^ into or out of cells. NKCC1 is an intensively studied member of the CCC family and plays fundamental roles in regulating *trans*-epithelial ion movement, cell volume, chloride homeostasis and neuronal excitability. Here, we report a cryo-EM structure of human NKCC1 captured in a partially loaded, inward-open state. NKCC1 assembles into a dimer, with the first ten transmembrane (TM) helices harboring the transport core and TM11-TM12 helices lining the dimer interface. TM1 and TM6 helices break α-helical geometry halfway across the lipid bilayer where ion binding sites are organized around these discontinuous regions. NKCC1 may harbor multiple extracellular entryways and intracellular exits, raising the possibility that K^+^, Na^+^, and Cl^−^ ions may traverse along their own routes for translocation. NKCC1 structure provides a blueprint for further probing structure–function relationships of NKCC1 and other CCCs.

## Abstract (web summary)

The human NKCC1 transporter belongs to the cation–chloride cotransporter (CCC) family and has important physiological functions, among them the regulation of chloride homeostasis and neuronal excitability. Here the authors present the 3.46 Å cryo-EM structure of human NKCC1 in a in a partially loaded, inward-open state and discuss the transport mechanism.

## Introduction

Cation–chloride cotransporters (CCCs) are secondary active symporters that harness the K^+^ and/or Na^+^ gradients generated by the Na^+^-K^+^-ATPase to extrude or uptake Cl^−^ in various cell types^1–5^. CCCs consist of two branches: three Na^+^-dependent Na^+^-(K^+^)-Cl^−^ (NCC and NKCC1-2) and four Na^+^-independent K^+^-Cl^−^ (KCC1–4) cotransporters. NKCC1 is one of the most extensively studied members of the CCC family and plays pivotal roles in *trans*-epithelial ion absorption and secretion, cell volume regulation, and regulation of chloride homeostasis and neuronal excitability, among others^6^. In most cell types, NKCC1 regulates cell volume in response to hypertonic stress as NKCC1-mediated Cl^−^ uptake and concomitant obligatory water influx represent a major defense mechanism against cell shrinkage. In polarized secretory epithelia cells, NKCC1 is highly expressed in the basolateral membrane where it absorbs Cl^−^ that is then secreted via other apically localized transport proteins (e.g., CFTR). In periphery sensory neurons and postnatal immature neurons, NKCC1 activity contributes to maintaining elevated [Cl^−^]_i_ such that GABA (**γ**-aminobutyric acid), a principle inhibitory neurotransmitter, stimulates Cl^−^ efflux and therefore, produces depolarizing and excitatory inward currents in these neurons. Given its role as a principle neuronal Cl^−^ accumulator that affects polarity of GABA responses, NKCC1 is an emerging target for developing therapeutics to treat neonatal seizures, neuropathic pain, and other neurological disorders^7–12^.

In the kidneys, NKCC2 and NCC are primary transport proteins involved in salt reabsorption in the apical membrane of the thick ascending limb of Henle’s loop and distal convoluted tubule of nephrons, respectively. Loss-of-function mutations of NKCC2 and NCC, respectively, lead to Bartter type I and Gitelman syndrome characterized by salt wasting and decreased blood pressure due to attenuated salt reabsorption^13–15^. Conversely, abnormally enhanced activity of NCC has been linked to salt-sensitive hypertensive disorders such as Gordon disease^16–18^. Given their fundamental roles in regulating blood volume and pressure, NKCC2 and NCC are attractive targets for developing novel anti-hypertensive therapeutics. Indeed, the loop and thiazide-type diuretics directly inhibit NKCC2 and NCC, respectively, and have been widely prescribed to treat hypertension and edema^19,20^.

CCCs belong to the large amino acid-polyamine-organocation (APC) superfamily of transporters. Structures of several APC transporters demonstrate that they all exhibit a rough twofold symmetry such that two inverted repeats of five transmembrane (TM) helices together form a central substrate binding cavity^21,22^. This pseudo-symmetric topology of two inverted repeats is the linchpin of the alternating-access mechanism whereby transporters isomerize among outward-open, occluded, and inward-open states to translocate substrates across membranes^23^. Hydropathy plot analyses and structure modeling based on related APC transporter structures predict that all CCCs share a common structural architecture with twelve TM helices harboring a 5 + 5 inverted repeat fold flanked by cytoplasmic N- and C-terminal domains^1,24,25^. CCCs are homo- or hetero-dimers and their C-terminal domains contribute to dimer assembly^26–30^. Despite a rudimentary functional map emerged from numerous mutagenesis studies of CCCs, essential structural features that underlie substrate binding and translocation, oligomeric assembly, inhibition by diuretics, and allosteric regulation by phosphorylation remain lacking.

Here, we report a cryo-EM structure of human NKCC1 captured in a partially loaded, inward-open state. NKCC1 assembles into a dimer partly via an interface formed by TM11–TM12 helices within the membrane bilayer. TM1 and TM6 helices lie at the core of the transport pathway with broken α-helical geometry roughly halfway across the lipid bilayer where two strictly conserved glycine residues may serve as flexible hinges that could facilitate the transporter to isomerize among different functional states. Ion binding sites are organized around these hinge regions with partially charged side chains, main chain atoms, and helix dipoles playing crucial roles in coordinating ions. In contrast to LeuT and many other APC transporters, NKCC1 seems to harbor multiple extracellular entryways and intracellular exits that converge at central ion binding sites, raising the possibility that K^+^, Na^+^, and Cl^−^ ions may traverse along their own routes for translocation. Our human NKCC1 structure provides a blueprint for further probing structure–function relationships of NKCC1 and other CCCs.

## Results

### Structure determination

**Fig. 1:**
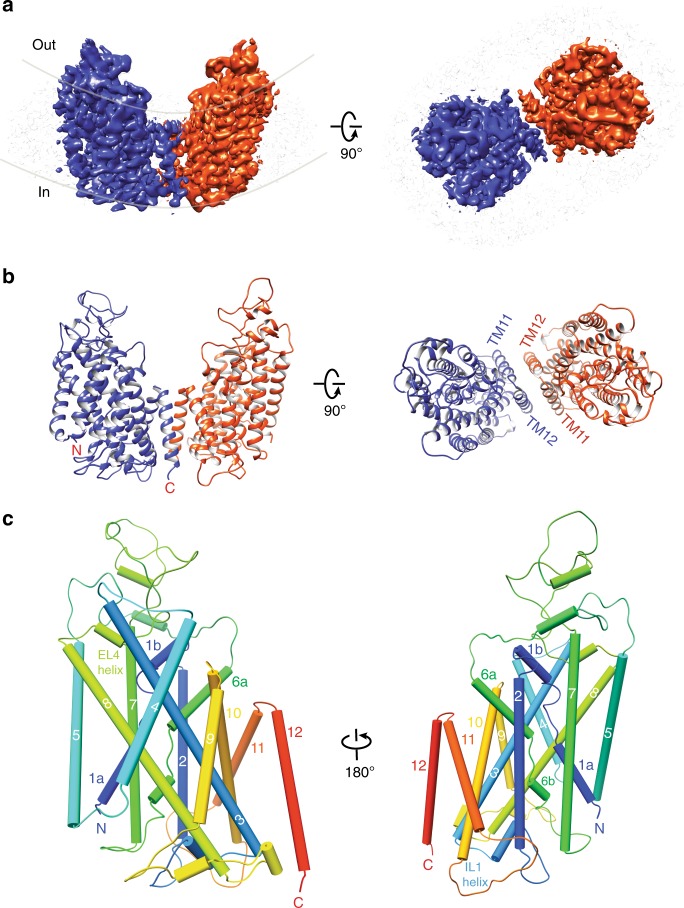
Human NKCC1 structure determined by single-particle cryo-EM. **a** Side and extracellular views of NKCC1 embedded in detergent micelles. Individual transporter subunits are color-coded. Densities of the detergent micelle are rendered semi-transparent and shown in light gray. Two lines (light gray) denote the outer and inner membrane layer, highlighting the curve architecture of NKCC1 dimer. **b** Ribbon representations of NKCC1 dimer are shown in same orientations and colored-coded as in panel (**a**). **c** An NKCC1 subunit is shown in cylinders with rainbow colors from the N- to C-termini.

We identified a human NKCC1 construct that eluted as a monodisperse peak on a size exclusion column and yielded homogenous particles suitable for 3D reconstruction by single-particle cryo-EM (Supplementary Fig. 1A). This construct bears deletions in two regions (255–278 within the cytoplasmic N-terminus; 941–1000 within the cytoplasmic C-terminal domain) that are divergent among Na^+^-dependent CCCs, as well as two single mutations (K289N and G351R). K289N mutation was found to enhance protein expression and G351R is a loss-of-function variant that was cloned from MDCK-LK-A3 cell line rendered deficient in co-transporting Na^+^–K^+^–2Cl^−^ ions by random mutagenization^31^. We performed Tl^+^ ion flux assay and found that NKCC1 bearing K289N mutation showed bumetanide (an inhibitory loop diuretic)-sensitive transport activity comparable to the wild-type NKCC1, and additional inclusion of the two above mentioned deletions resulted in a transporter with no statistically significant transport activity. As expected, our cryo-EM construct that further bears the G351R loss-of-function mutation showed complete loss of transport function, as compared with the wild-type NKCC1 (Supplementary Fig. 1B-E). We speculated that this NKCC1 construct may be arrested in a specific state along the transport cycle, and thus could be more conformationally homogeneous for structural analyses. NKCC1 transport activity is enhanced by phosphorylation at several conserved Ser/Thr residues at its N-terminus likely due to increased structural dynamics of its large cytoplasmic C-terminal domains^30,32–35^. To further minimize structural flexibility, we co-expressed the NKCC1 construct with phosphatase PP1α and treated purified transporter protein with recombinant PP1α to promote a fully de-phosphorylated (inactive) transport state^36^. We also excluded K^+^ ion, a substrate that NKCC1 translocates, during sample preparation, which we speculated may prevent NKCC1 from completing a full transport cycle and further arrest the transporter in a specific state. We determined human NKCC1 transporter structure at 3.46 Å resolution that allows for de novo model building (Fig. 1; Supplementary Figs. 2, 3, and 4). The final map shows well-resolved densities for the transmembrane domain, large extracellular domains, and loops connecting membrane-spanning helices; however, it lacks interpretable densities for the cytosolic domains, consistent with reference free 2D class averages that show merely a cloud of fuzz densities for the cytosolic domains (Supplementary Fig. 2). A recently reported *Danio rerio* NKCC1 structure successfully resolved the cytoplasmic C-terminal domains^37^, whereas the human KCC1 structures also failed to resolve the C-terminal domains^38^.

### Dimeric architecture of the human NKCC1 transporter

As expected from previous biochemical crosslinking, FRET (fluorescence resonance energy transfer), and structural analyses^26–30,37^, NKCC1 assembles as a homodimer, exhibiting twofold symmetry along an axis perpendicular to the membrane (Fig. 1a, b). Overall, our human NKCC1 structure largely superimposes with that of *Danio rerio* NKCC1, with major differences residing at their extracellular domains and loops collecting transmembrane helices (Supplementary Fig. 5A). Of note, human NKCC1 and KCC1 adopt a drastically different dimeric architecture, although a single subunit of these transporters superimposes nicely within the transmembrane region (Supplementary Fig. 5B). Each NKCC1 monomer consists of twelve membrane-spanning helices with the first five helices (TM1–TM5) related to the next five helices (TM6–10) by a pseudo twofold symmetry axis parallel to the membrane. The remaining TM11–TM12 helices adopt an inverted V-shaped helix-turn-helix structure and associate with the same structure from a second subunit, constituting the primary dimerization interface within the lipid bilayer in addition to that formed by the cytoplasmic C-terminal domains (Fig. 1b). Notably, in contrast to significant conservation (~64% identical) within their TM1–TM10 helices, NKCC1, NKCC2, and NCC exhibit only modest sequence identity (~38%) within their TM11–TM12 helices, providing a plausible explanation for lack of heteromeric dimers among these transporters (Supplementary Fig. 6). Mismatches within the dimeric interface of their cytoplasmic C-terminal domains may represent another mechanism that heteromeric dimers are disfavored within the Na^+^-dependent clade of CCCs^27^. On the contrary, four KCC (KCC1–4) transporters bear almost identical sequences in their TM11–TM12 helices and thus are expected to form complementary dimer interfaces within the lipid bilayer. Indeed, KCC isoforms assemble into various heteromeric transporters when co-expressed in heterologous host cells^28^. Beside these major transmembrane helices, an extracellular loop (EL)-4 helix preceding TM8 and an intracellular loop (IL)-1 helix between TM2 and TM3 lie almost parallel to and are in close contact with the outer and inner leaflets of the bilayer, respectively (Fig. 1c). These two short helices are related by the internal pseudo symmetry and seem to be strategically positioned to affect the extracellular and intracellular gates, respectively.

Interestingly, the NKCC1 transmembrane core adopts a conspicuously curved architecture because TM helices located at the periphery of the dimer are gradually elevated toward the extracellular side as compared with those residing at the center of the dimer (Fig. 1a, b). Indeed, the most periphery helices (TM4–TM5) are situated ~9 Å above the central helices (TM11–TM12). This curved dimer raises the possibility that the lipid bilayer might be deformed at regions where NKCC1 transporter density (and activity) is substantial (e.g., basolateral membrane of secretory epithelia) and that membrane fluidity could regulate NKCC1 transport activity.

### Extracellular domains

**Fig. 2:**
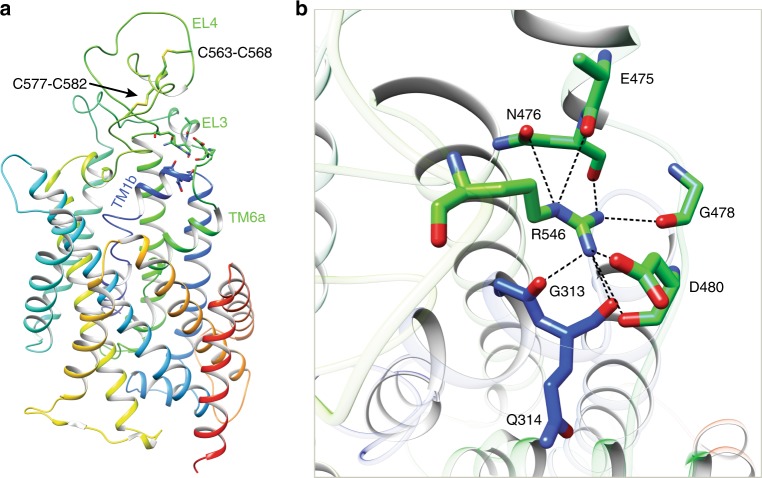
Extracellular domain of NKCC1 interacts with the transmembrane core. **a** A NKCC1 subunit is shown in ribbon diagram with rainbow colors from N- to C-termini. Two disulfide bonds within EL4 are shown as sticks. Residues that mediate contacts among EL3, EL4, and TM1b are also depicted in sticks. **b** A zoomed view highlights interactions among EL3, EL4, and TM1b. Interactions are denoted with dashed black lines.

Above the transmembrane core of NKCC1, two large extracellular loops (EL3 between TM5 and TM6; EL4 between TM7 and TM8) fold into ordered structures that sit atop and interact extensively with the transmembrane core (Fig. 2a). Of note, EL4 is stabilized by two disulfide bonds (Cys563–Cys568 and Cys577–Cys582; Fig. 2a), consistent with a previous finding that these four cysteine residues do not react with reagents that modify free -SH group^39^. Notably, substitution of C421 in NCC (equivalent to Cys568 in human NKCC1) to Arg causes Giltelman’s disease in human possibly by disrupting this essential stabilizing disulfide bridge^40^. Interestingly, in the *Danio rerio* NKCC1 structure, only a single disulfide bond equivalent to Cys577–Cys582 in human NKCC1 was observed; the other pair of cysteines (equivalent to Cys563 and Cys568 in human NKCC1) are in close proximity, but appear not to form a covalent bond^37^. This subtle difference likely reflects the fact that the EL4 loop is one of the most divergent segments among various NKCC1 homologs.

One particular interesting interaction network involves the extracellular end of TM1, a C-terminal segment of EL3 preceding TM6, and an EL4 segment immediately following TM7 (Fig. 2b). Here, Arg546 residing at EL4 makes extensive hydrophilic contacts with main chain carbonyl oxygens from residues Gly313, Gln314, Glu475, and Gly478, as well as side chains of Asn476 and Asp480. In addition, Arg546 possibly also neutralizes the negative dipole existing at the C-termini of TM1 helix. Importantly, this arginine residue is invariant in all CCCs and mutation of this Arg to Cys in NCC (R399C) results in Giltelman’s syndrome, highlighting the importance of this interaction network in supporting transport function^40^. Although EL3 and EL4 of NKCC1, NKCC2, and NCC share several highly conserved motifs, these extracellular structures are by far more divergent in primary sequences than transmembrane helices. Future studies will determine whether this sequence diversity can be exploited to target these functionally important extracellular structures for developing isoform-specific pharmacological agents to treat various human diseases.

### Ion transport pathways

**Fig. 3:**
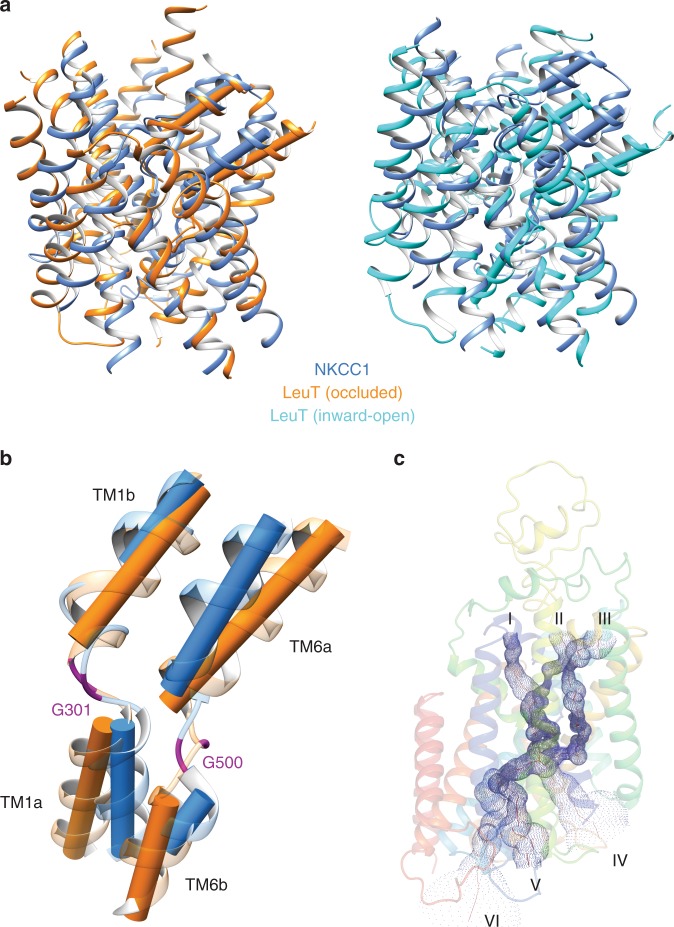
NKCC1 structure in an inward-open state. **a** Superimposition of the first ten TM helices of human NKCC1 with those of LeuT in occluded state (left) or in inward-open state (right). Structures are shown in ribbon diagram. TM1 and TM6 helices, which undergo significant conformational changes among different transport states, are also depicted as cylinders. **b** Comparison of TM1 and TM6 helices in NKCC1 and LeuT (occluded). The glycine residues in the flexible hinge regions are highlighted with magenta color. **c** Solvent accessible pathways in NKCC1 are mapped with HOLE. Three entryways, three exits, and central cavity are outlined with blue dots.

From a bird’s eye view, NKCC1 shares many structural hallmarks in its substrates (i.e., ions) transport pathway with other APC transporters, such as LeuT that mediates Na^+^-coupled leucine uptake in *Aquifex aeolicus*^41^, in line with previous modeling and mutagenesis studies^24^. LeuT arguably represents the best understood APC transporter with respect to its transport mechanisms due in no small part to insights gained by a series of LeuT structures captured in outward-open, occluded, or inward-open states^42,43^. We therefore superimposed TM1–TM10 helices of our human NKCC1 structure onto each of these LeuT structures and found that NKCC1 structure aligns best with that of the occluded state of LeuT (Fig. 3a, b); TM11 and TM12 helices were excluded in these comparisons because they are two optional helices in APC transporters and, when present, tend to have diverse structural and functional roles in different transporters^44^. There was one important caveat in such comparisons because NKCC1 transports K^+^, Cl^−^, and Na^+^ ions, all of which are significantly smaller in size than LeuT substrate leucine. We therefore mapped potential membrane-embedded solvent accessible channels using program HOLE^45^. We found that three potential extracellular entryways (I, II, and III) lead to central ion binding sites and that three putative intracellular conduits (IV, V, and VI) would allow ions to exit central sites into cytoplasm (Fig. 3c). These ion pathways are primarily formed by TM1, TM2, TM3, TM6, TM8, and TM10 helices that are rich in partially charged hydrophilic residues (e.g., Ser, Thr, Asn, and Tyr), but lack formal charged residues (e.g., Glu and Asp). Such arrangement likely reflects a functional requirement because Glu and Asp residues tend to interact strongly with ions and thus could impede ion transport. Indeed, merely replacing Glu290 to Ser in LeuT establishes a new Cl^−^ binding site along the translocation pathway and converts LeuT into a Na^+^/Cl^−^ dependent transporter^46,47^. All three extracellular entryways are constricted, with the shortest distances between opposing side chains being 3.3 Å in entryway I, 4.3 Å in entryway II, and 4.9 Å in entryway III, and appear to be impermeable to hydrated K^+^ (6.0 Å), Cl^−^ (5.8 Å), or Na^+^ (6.6 Å) ions (Fig. 3c). In contrast, all three intracellular pathways are considerably wider than entryways and comparable to ion permeation pathways seen in open ion channels. In particular, the pathway IV, a vestibule formed by TM1, TM6, TM5, and TM8, is sufficiently wide for unobstructed flow of fully hydrated ions once they dissociate from their ruptured central binding sites. Of note, this widest intracellular pathway is topological equivalent to an intracellular vestibule observed in an inward-open LeuT through which leucine exits central substrate binding site^43^. We therefore concluded that our NKCC1 structure is likely trapped in an inward-open state.

### Central ion binding sites

**Fig. 4:**
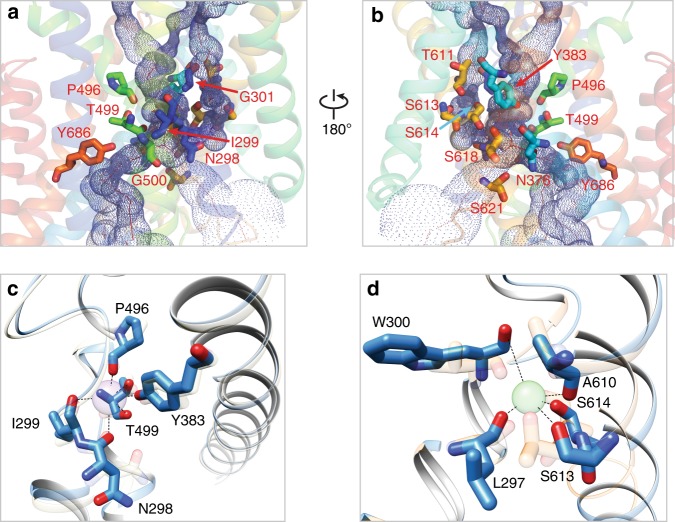
Central ion binding sites in human NKCC1. **a**, **b** Residues directly involved in ion coordination and hydrophilic residues residing in close proximity to ion binding sites are shown in sticks. **c** Comparison of human and *Danio rerio* NKCC1 K^+^ binding site. K^+^ coordinating residues are shown in sticks. K^+^ (sphere) and equivalent residues in *Danio rerio* NKCC1 are rendered as semi-transparent sticks. **d** Comparison of the putative human NKCC1 Na^+^ site with the Na^+^ site 2 in LeuT. Na^+^ coordinating residues in NKCC1 are shown as sticks. Na^+^ (sphere) and equivalent residues in LeuT are rendered as semi-transparent sticks.

As first discovered in LeuT^42^, TM1 and TM6 helices of NKCC1 also become discontinuous roughly at the middle of the membrane bilayer and are separated into four half helices, with two glycine residues residing at the hinge regions (Gly301 between TM1a and TM1b; Gly500 between TM6a and TM6b; Fig. 3b). These two glycine residues are strictly conserved in all CCCs and may function as two flexing points around which four half helices can bend to facilitate isomerization of transporters among different states, as observed in LeuT^43^. Indeed, a loss-of-function G193R variant of NKCC2 (equivalent to Gly301 in human NKCC1) results in Bartter’s disease, highlighting the functional importance of bearing a flexible glycine in this hinge region^48^. Similar to LeuT, ion binding sites in NKCC1 are also organized around these two hinge regions, with nearby hydrophilic side chains, main chain atoms, and helix dipoles playing key roles in ion coordination (Fig. 4)^42^.

We intentionally excluded K^+^ from our sample in order to arrest NKCC1 in a specific transport state and accordingly did not observe K^+^ density in our map. A recent *Danio rerio* NKCC1 structure was determined using a sample containing K^+^ ion and resolved K^+^ binding site^37^. Much to our surprise, human and *Danio rerio* NKCC1 structures essentially superimpose at the K^+^ binding site, indicating that lack of K^+^ coordination alone does not lead to rupture of the K^+^ binding site possibly because it is structurally coupled to and stabilized by nearby Na^+^ and Cl^−^ binding sites. A similar finding was recently discovered in human KCC1 where exclusion of K^+^ also does not appreciably perturb the structure of the transporter^38^. In human NKCC1, the K^+^ ion binding site comprises of exclusively partial charges, including main chain carbonyl oxygen atoms of Asn298 (TM1), Ile299 (TM1), Thr499 (TM6), and Pro496 (TM6), as well as hydroxy groups of Tyr383 (TM3) and Thr499 (Fig. 4c). Tyr383 plays a crucial role in ion transport possibly by defining K^+^ specificity, as even a conserved substitution of this Tyr to Phe renders human NKCC1 inactive^49^. Of note, the NCC transporter conspicuously bears a histidine (H234) at this position (Supplementary Fig. 6), providing a plausible explanation for its K^+^-independent cotransport of only Na^+^ and Cl^−^ ions as noted previously^37^. Intriguingly, all the four remaining K^+^ coordination residues are strictly conserved in NCC, making us wonder whether substitution of this Tyr to His could transform a K^+^ binding site in NKCC1, NKCC2, and KCCs into perhaps a Cl^−^ binding site. Indeed, the N–H on histidine imidazol group frequently serves as a hydrogen bond donor in coordinating Cl^−^ ion in other Cl^−^ binding proteins^50^. Irrespective of whether this histidine and other four residues participate in Cl^−^ binding in NCC, they are certainly crucial for NCC function as mutations in two of these residues (H234Q and P349L) are associated with Giltelman’s disease^40^.

Our human NKCC1 map lacks non-protein densities that could be ascribed to Na^+^ ion as does the recently reported *Danio rerio* NKCC1 structure^37^. However, NKCC1 exhibits remarkable conservation with LeuT at a Na^+^ binding site commonly referred to as Na^+^ site 2 in APC transporters and bears all the structural and chemical hallmarks essential for Na^+^ coordination^42^. In human NKCC1, this putative Na^+^ binding site consists of residues residing on TM1 and TM8 helices, including Leu297 and Trp300 on TM1 and Ala610, Ser613, and Ser614 on TM8 (Fig. 4d). Similar to the K^+^ binding site, the Na^+^ binding site involves main chain oxygen atoms and hydroxyl groups of two serine residues, without contribution from any formal charged residues. Remarkably, these five residues are invariant in NKCC1, NKCC2, and NCC, likely reflecting a stringent functional requirement for all Na^+^-dependent CCCs. Indeed, perturbations of this putative Na^+^ binding site by genetic mutations lead to human diseases, including an NCC A467T variant associated with the Giltelman’s disease and an NKCC2 S507P variant found in Bartter’s disease patients^40,48^. The putative NKCC1 Na^+^ binding site also aligns with the Na^+^ site 2 in the SiaT transporter as previously noted^37^, further corroborating that these five residues constitute a Na^+^ binding site in NKCC1 and perhaps in NKCC2 and NCC as well. Of note, the recently reported human KCC1 structures lack this putative Na^+^ binding site, lending further evidence to support the presence of Na^+^ in this site^38^.

### The extracellular gates and entryways

**Fig. 5:**
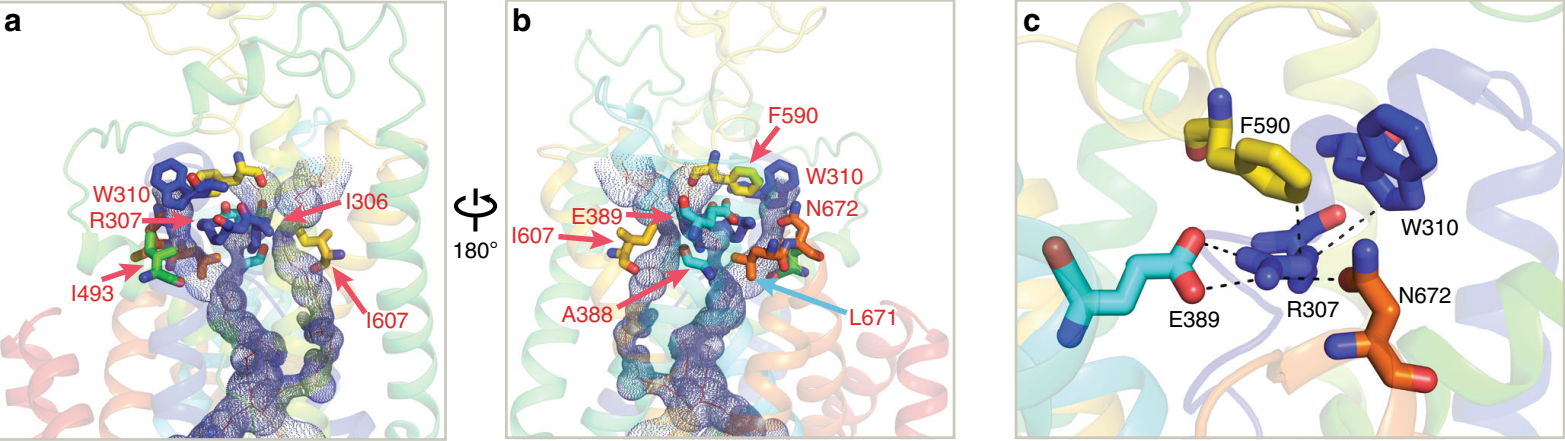
Extracellular entryways and gates in NKCC1. **a**, **b** Residues involved in closing extracellular entryways are shown in sticks. **c** Gating interactions in the extracellular entryways. Residues that participate in gating interactions are shown in sticks and interactions between these residues are denoted with black dashed lines.

Our human NKCC1 structure showed closed extracellular gates as all three potential solvent accessible entryways leading to central ion binding sites are too constricted to permit hydrated K^+^, Na^+^, and Cl^−^ ions to flow through. TM1b, TM6a, extracellular ends of TM3 and TM10, and a short EL4 helix preceding TM8 are in close proximity and together form impermeable barriers for hydrated ions just beneath the outer leaflet of the membrane bilayer (Fig. 5a, b). These barriers (or gates) are likely stabilized by a multitude of hydrophobic, hydrogen bonding, and ionic bonding interactions that involve a set of conserved residues. One most extensive interaction hub involves Arg307 and Trp310 on TM1a, Glu389 on TM3, Phe590 on an EL4 helix, and Asn672 on TM10, offering an immediate impression that these hydrophilic interactions may staple these structural elements together and hence force closure of the extracellular gates (Fig. 5c). In particular, Arg307 appears to play a central role in coordinating this interaction hub as it interacts with Glu389, Asn672, and Phe590 via salt bridge, hydrogen bond, and cation-π, respectively. Several clinical mutations likely affect the overall structure and/or conformational dynamics of the extracellular gates, including two NCC variants (R158Q and R158L) found in patients with Giltelman’s disease and three NKCC2 variants (R199C, R199G, and W202C) associated with Bartter’s syndrome^40,48^. The fact that substitutions of this arginine to four amino acids of different sizes and chemical properties in NCC and NKCC2 are all pathogenic underscores a potentially conserved role for this arginine residue in closing extracellular gates that represents an essential step along a transport cycle for any solute carrier transporters. This arginine residue is also conserved in all KCCs, suggesting that similar gating interactions may operate in these transporters as well. Even more remarkably, in LeuT, an equivalent arginine residue interacts with an aspartate residue via a salt bridge, which contributes to closing an extracellular gate to allow the transporter to isomerize into an inward-open state^43^.

### The intracellular pathways and opened intracellular gates

**Fig. 6:**
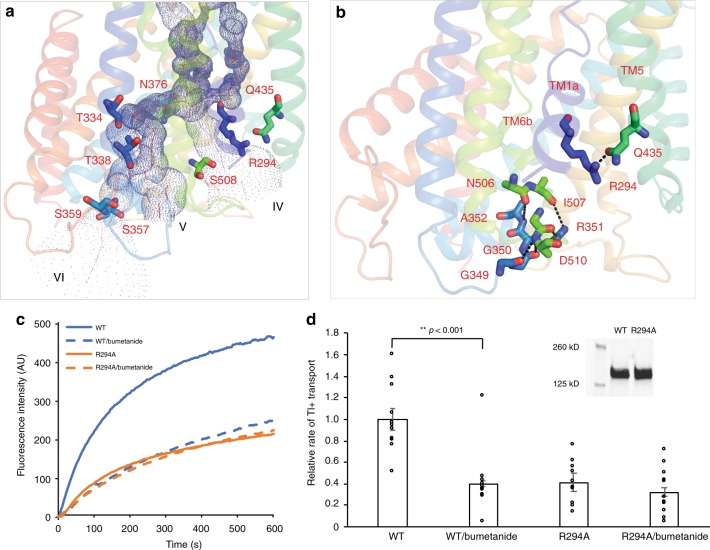
Intracellular vestibules and interactions stabilizing inward-open conformation. **a** Hydrophilic residues lining the intracellular exits are shown in sticks. **b** Residues that participate in stabilizing inward-open conformation are shown in sticks. Interactions are denoted with black dashed lines. **c** Kinetics of transport were measured for the WT NKCC1 and R294A mutant using Tl^+^ ion flux assay. **d** Rates of transport were all normalized to that measured for the WT NKCC1 without inhibition by bumetanide. Each circle represents a kinetic measurement of a single sample and error bars represent s.e.m. The R294A mutant shows no statistically significant bumetanide-sensitive transport. The inset shows that the WT NKCC1 and R294A mutant exhibited similar expression level as determined by western blot.

Our human NKCC1 structure showed three intracellular vestibules (IV, V, and VI) that converge at a central cavity located halfway across membrane where three ions bind to their respective sites. All of these three intracellular pathways are notably wider than the three entryways and would afford unobstructed conduits for ions to reach cytoplasm once they dissociate from their central binding sites. The three intracellular vestibules are lined by a multitude of hydrophilic residues which may be essential for maintaining these pathways in a hydrated state for efficient ion translocation (Fig. 6a). This has been previously shown in ion channels that a hydrophobic pore with a diameter <14 Å can spontaneously de-wet (or lose water) and prevent ion conduction^51^. For instance, the intracellular vestibule V is primarily formed by TM2 and a short helix on intracellular loop 1 (IL1), with three hydrophilic residues (Thr334, Thr338, and Ser359) pointing their hydroxy groups toward the center of the ion pathway (Fig. 6a). Interestingly, human NKCC2 has three splicing isoforms (A, B, and F) that differ only in TM2 and IL1, yet exhibit different affinities for ions^52,53^. Although conversions of many isoform-specific residues within this region, including two hydrophobic residues, accordingly convert ion affinity profiles, Thr249 (equivalent to Thr338 in NKCC1) appears to have the largest effect on Cl^−^ affinity^54,55^. Moreover, in NKCC1, substitution of Asn376, which resides just beneath central ion binding sites, to cysteine reduces affinity to all three ions, whereas N376W mutant completely loses transport function possibly because the bulky tryptophan may plug the ion pathway^49^.

Our human NKCC1 structure is trapped in an inward-open state, so residues and interactions that would close intracellular gates in outward-open or occluded states await future structural and functional analyses. Here, we focus on understanding how intracellular gates are stabilized in an open configuration in the inward-open state. The entryway of the widest intracellular vestibule (or pathway IV) is surrounded by TM1a, TM6b, TM5, and TM8 (Fig. 6b), among which TM1a and TM6b may be the most mobile structural elements as they could undergo hinge-bending movements around two hinges located halfway across the lipid bilayer as seen in LeuT^43^. A stable open intracellular gate conceivably involves formation of new gating interactions that would immobilize TM1a and TM6b. Indeed, in our NKCC1 structure, TM1a is anchored to TM5 by hydrogen bonding interactions between Arg294 (TM1a) and Gln435 (TM5). R294A mutation resulted in reduced transport activity as measured by Tl^+^ ion flux assay when compared with the wild-type NKCC1 (Fig. 6c, d). In addition, substitution of R145 (equivalent to Arg294 in human NKCC1) to cysteine in NCC results in Giltelman’s disease^40^. Together, these observations suggested a key gating role for this conserved arginine in Na^+^-dependent CCCs. TM6b also appears to be constrained as the cytoplasmic end of TM6b and the intracellular loop (IL3) immediately following it are in close contact with IL1 (^349^GGGA^352^) via hydrogen bonding interactions. We used a loss-of-function G351R variant of NKCC1 for structure determination and interestingly^31^, this arginine residue establishes additional hydrogen bonding interactions with TM6a and IL3 (Fig. 6b). Notably, a similar mutation (G243D) in NKCC2 causes mild Bartter’s disease^54^ and it remains to be determined whether this aspartate can also engage in hydrogen bonding interactions as does arginine in the NKCC1 G351R variant.

## Discussion

NKCC1 shares similar structural design principles with LeuT and many other APC transporters, including TM1 and TM6 helices being discontinuous at the middle of the lipid bilayer and substrate binding sites centered around these discontinuous hinge regions. However, NKCC1 unusually has multiple extracellular entryways and intracellular exits that converge at central ion binding sites located approximately halfway across the membrane bilayer. Such design of multiple translocation pathways could simply reflect that ions shuttled by NKCC1 are much smaller in size than leucine and other bulky substrates translocated by other APC transporters, so these pathways could just be redundant. A more provocative explanation, though, is that Na^+^, K^+^, and Cl^−^ ions need traverse along separate routes to cross the membrane bilayer. Of note, three negatively charged residues, Glu429 and Glu431 located on TM5 and Asp510 located on IL3, surround the intracellular opening of the vestibule IV, offering an impression that such electronegativity would attract and facilitate conduction of K^+^ and Na^+^, but not Cl^−^, as first seen in KcsA and later in many other cation channels as well^56^. Another important structural trait of NKCC1 is that it may undergo more subtle conformational changes than other APC transporters as these transporters proceed along the transport cycle. Indeed, our NKCC1 structure is captured in an inward-open state, but aligns best with the occluded structure of LeuT, as intracellular vestibules that are sufficiently wide for ions to reach central binding sites in NKCC1 would block bulky substrates such as leucine. Subtle conformational changes required for alternating among different functional states in NKCC1 may also explain why it exhibits much faster turnover rate (~3500/s at 37 °C) than other APC transporters as previously noted^49^.

Human and *Danio rerio* NKCC1 structures were determined in buffer containing two substrate ions Na^+^ and Cl^−^ or all three substrate ions, respectively, so they are expected to be captured in two distinct substrate bound states, yet these two structures essentially superimpose and are all trapped in an inward-open conformation. In a recent kinetic transport model of NKCC1^57^, inward-open transporter first binds a Cl^−^, then a Na^+^, followed by a second Cl^−^ and a K^+^. This model further proposes that binding of Na^+^ and Cl^−^, or of K^+^ and Cl^−^ are strictly coupled, so eletroneutral ion transport is guaranteed. This model also allows incomplete transport reaction to occur whereby a partially loaded transporter (e.g., loaded only with Na^+^ and Cl^−^) changes conformation and opens to the opposite site of membrane, resulting in for example K^+^/K^+^ exchange, instead of unidirectional transport of Na^+^–K^+^–2Cl^−^. Using this kinetic model as a reference, our human NKCC1 structure would represent an inward-open transporter partially loaded with a Na^+^ and a Cl^−^ and arrested on route before binding K^+^ and Cl^−^ because K^+^ was excluded in sample preparation, whereas the *Danio rerio* NKCC1 structure represents an inward-open transporter fully loaded with K^+^, Na^+^, and Cl^−^ ions. We showed that the partially loaded, inward-open NKCC1 transporter is thermodynamically stable and surprisingly resembles a fully loaded transporter in structure. It is conceivable that this partially loaded NKCC1 transporter could isomerize into outward-open conformation and if true, then can account for experimental observations that NKCC1 can function partially to mediate K^+^/K^+^ exchange in several cell types and physiological conditions^12,58,59^. Our human NKCC1 structure, together with zebrafish NKCC1 and human KCC1 structures^37,38^, now provides a blueprint for further probing structure–function relationships of NKCC1 and other CCCs.

## Methods

### Molecular biology

Primers for cloning all expression constructs of human NKCC1 used in this study are listed in Supplementary Table 1.

### Expression and purification of human NKCC1

The human NKCC1 construct, encompassing residues 2–1212 and bearing deletions in two regions (255–278 within the cytoplasmic N-terminus; 941–1000 within the cytoplasmic C-terminal domain) and two single mutations (K289N and G351R), was expressed with an N-terminal maltose binding protein (MBP) fusion in HEK293S GnTI^−^ (ATCC CRL-3022) cells using the BacMam system as described^60^. Human PP1α (2–330) also with an N-terminal MBP fusion was co-expressed with NKCC1 to de-phosphorylate transporters within cells. In brief, HEK293S GnTI^−/−^ cells, grown in suspension in freestyle 293 expression medium (Invitrogen, Carlsbad, CA) at 37 °C in an orbital shaker, were transduced with NKCC1 and PP1α baculoviruses when cell density reached ~2 × 10^6^/ml. Eight to twenty-four hours post transduction, sodium butyrate was added to the culture to a final concentration of 5 mM to enhance protein expression; temperature was reduced to 30 °C. Cells were harvested 72 h post transduction for subsequent affinity purification with amylose resin (New England BioLabs, Ipswich, MA). Human NKCC1 protein was eluted from amylose resin with buffer composed of (in mM) 50 HEPES (pH 7.4), 150 NaCl, 0.5 TCEP, 0.5 MNG-3, 0.1 CHS, 1 MnCl_2_, 20 maltose, 0.1 mg/ml soybean lipids, 10% glycerol. Recombinant PP1α was then added to purified NKCC1 proteins for further de-phosphorylation overnight and MBP tag was simultaneously removed by protease cleavage. NKCC1 transporter was separated with a Superose 6 column using buffer composed of (in mM) 20 HEPES (pH 7.4), 150 NaCl, 0.5 TCEP, 25 × 10^−3^ MNG-3, and 5 × 10^−3^ CHS, and peaks corresponding to NKCC1 were pooled and concentrated for cryo-EM analyses.

### Thallium ion (Tl^+^) flux transport assay

The NKCC1-mediated K^+^ flux was measured using Tl^+^ as a surrogate ion according to previously published protocols with slight modifications^61^. Briefly, the wild-type human NKCC1 and three mutants were expressed in HEK293T cells (ATCC CRL-3216) as an *N*-terminal YFP fusion proteins using BacMam expression system that typically reached peak YFP fluorescence in 48 h post transduction. Expression of R294A and wild-type NKCC1 was also confirmed by western blot using mouse anti-GFP antibody (Roche, Cat. No. 11,814,460,001; 1:1000 dilution). For transport assay, ~1.25 × 10^5^ HEK293T cells were seeded per well in a 96-well plate 1 day prior to baculovirus transduction, and sodium butyrate was added to each well at 5 mM final concentration 8–12 h post transduction to boost transporter expression. Transport assay was performed between 36 and 60 h post transduction. Growth DMEM media was replaced with 80 µL of low Cl^−^ buffer (15 mM Na-HEPES, pH 7.4, 135 mM Na-gluconate, 1 mM MgCl_2_–6H_2_O, 1 mM Na_2_SO_4_, 1 mM CaCl_2_–2H_2_O) supplemented with FluxOR™ Red dye, power load reagent and probenecid provided in the FluxOR-Red Potassium Ion Channel Assay kit (Thermo Fisher Scientific), and cells were incubated at 37 °C for 1 h. Dye-loaded cells were then washed with the same low Cl^−^ buffer mentioned above or washed with low Cl^−^ buffer supplemented with 33 µM bumetanide. Fluorescence intensity was measured on a Synergy Neo2 HTS Multi-Mode Microplate Reader (BioTek) using bottom read mode with 4.5 mm read height. Excitation wavelength (540 nm) and emission wavelength (590 nm) were controlled by monochromator. The transport assay was initiated by adding 20 μl of 12 mM thallium sulfate (6× concentrated in low Cl^−^ buffer) to each well and the transport kinetics were followed every 3 s for at least 120 s. To increase reading accuracy, a 10-ms delay was applied after each plate movement and one data point represents an average of 255 measurements. The relative transport rate was approximated as the rate of fluorescent intensity change per second based on the maximum initial rate which represents the best linear fit of 10 data points (i.e., 30 s) with highest slope. Student *t* test was used to evaluate the significance of NKCC1-mediated, bumetanide-sensitive transport activity.

### Cryo-EM data acquisition

In all experiments, 3.5 μl of NKCC1 sample at ~3–5 mg/ml, supplemented with 50 µM furosemide, was applied to glow-discharged Au 1.2/1.3 holey, 300 mesh gold grids. Grids were plunge frozen in liquid ethane using a Vitrobot Mark III (FEI) set to 4 ˚C, 80% relative humidity, 20 s wait time, −1 mm offset, and 7 s blotting time. Data were collected on a Krios (FEI) operating at 300 kV equipped with the K2 Summit direct electron detector at the University of Utah, PNCC, NCCAT, and Yale University. Movies were recorded using SerialEM^62^, with a defocus range between −1.0 and −3.5 μm. Specifically, movies were recorded in super-resolution counting mode at a magnification of 47,619x, which corresponds to a physical pixel size of 1.05 Å. The data were collected at a dose rate of 1.56 e^−^/Å^2^/frame with a total exposure of 36 frames, giving a total dose of 56 e^−^/Å^2^.

### Image processing 3D reconstruction and model building

Movie frames were aligned, dose weighted, and then summed into a single micrograph using MotionCor2^63^. CTF parameters for micrographs were determined using the program CTFFIND4^64^. Approximately 2000 particles were manually boxed out in RELION to generate initial 2D averages, which were then used as template to automatically pick particles from all micrographs^65^. A total of 899,683 particles were extracted in RELION, and then imported into cryoSPARC for one round of 2D classification^66^. ‘Junk’ particles that were sorted into incoherent or poorly resolved classes were rejected from downstream analyses. The remaining 489,720 particles from well-resolved 2D classes were pooled, and were used to calculate a de novo model in cryoSPARC without imposing any symmetry. The initial model was further improved to ~8 Å by homogenous refinement in cryoSPARC with C2 symmetry imposed. Subsequently, two rounds of heterogenous 3D classification were performed in cryoSPARC without imposing any symmetry using the 8 Å map as initial model. After the second round 3D classification, a single class showing clear secondary structural features and best map connectivity was selected. The 107,589 particles in this class were subjected to non-uniform refinement in cryoSPARC and yielded a 3.8 Å map. At this point, the 107,589 particles were exported into RELION for 3D classification without alignment (i.e., the Euler angles and in plane translations calculated by non-uniform refinement in cryoSPARC were maintained). The best class contains 75,748 particles which were again imported back to cryoSPARC for non-uniform refinement that calculated a final map of 3.46 Å resolution when solvent noise and detergent micelle belt were masked out.

The 3.46 Å resolution map was locally sharpened in cryoSPARC with an overall B factor of ~−100 Å^2^ for model building in Coot^67^. The model was refined in real space using PHENIX^68^, and assessed in Molprobity (Supplementary Table 2)^69^. We could not identify any densities to account for the furosemide molecule and future studies are needed to understand the site(s) and mechanism of inhibition by this clinically important class of loop diuretics. FSC curves were then calculated between the refined model vs summed half maps generated in cryosparc, and resolution was reported as FSC = 0.5 (Supplementary Fig. 2). The directional resolutions were calculated using a remote 3DFSC processing server (https://3dfsc.salk.edu/)^70^. UCSF Chimera was used to visualize and segment density maps, and figures were generated using Chimera and Pymol. HOLE program was used to calculate solvent accessible pathways^45^.

### Reporting summary

Further information on research design is available in the Nature Research Reporting Summary linked to this article.

## Supplementary information

Supplementary Information

Reporting Summary

## Data Availability

The cyro-EM map of the human NKCC1 structure has been deposited in the Electron Microscopy Data Bank (EMDB) with the accession code EMD-20537. The atomic coordinate for the corresponding model has been deposited in the Protein Data Bank (PDB) with the accession code 6PZT. The source data underlying Fig. 6c, d and Supplementary Figs. 1B-E are provided as a Source Data file. All other data and reagents that support the findings of this study are available from the corresponding author upon reasonable request.

## Source Data

Source Data
