# Author Correction: Structure of the human cation-chloride cotransporter NKCC1 determined by single-particle electron cryo-microscopy

**DOI:** 10.1038/s41467-020-16303-8

**Published:** 2020-05-07

**Authors:** Xiaoyong Yang, Qinzhe Wang, Erhu Cao

**Affiliations:** Department of Biochemistry, University of Utah School of Medicine, Salt Lake City, UT 84112-5650 USA

**Keywords:** Biochemistry, Structural biology, Electron microscopy, Cryoelectron microscopy

Correction to: *Nature Communications* 10.1038/s41467-020-14790-3, published online 21 February 2020.

The original version of this Article omitted the following from the Acknowledgements: A portion of this research was supported by NIH grant U24GM129547 and performed at the PNCC at OHSU and accessed through EMSL (grid.436923.9), a DOE Office of Science User Facility sponsored by the Office of Biological and Environmental Research. Some of this work was performed at the National Center for CryoEM Access and Training (NCCAT) and the Simons Electron Microscopy Center located at the New York Structural Biology Center, supported by the NIH Common Fund Transformative High Resolution Cryo-Electron Microscopy program (U24 GM129539) and by grants from the Simons Foundation (SF349247) and NY State. This has now been corrected in both the PDF and HTML versions of the Article.

