# Supplementary material for: Structure of the human cation–chloride cotransporter NKCC1 determined by single-particle electron cryo-microscopy
Source: Nat Commun. 2020 Feb 21;11:1016. doi: 10.1038/s41467-020-14790-3 (PMC7035313; doi:10.1038/s41467-020-14790-3)
Supplement: Supplementary file 1 — Supplementary Information [file 41467_2020_14790_MOESM1_ESM.pdf]

Supplementary Information

Structure of the human cation-chloride cotransporter NKCC1 determined by single-particle electron cryo-microscopy

Xiaoyong Yang et al.

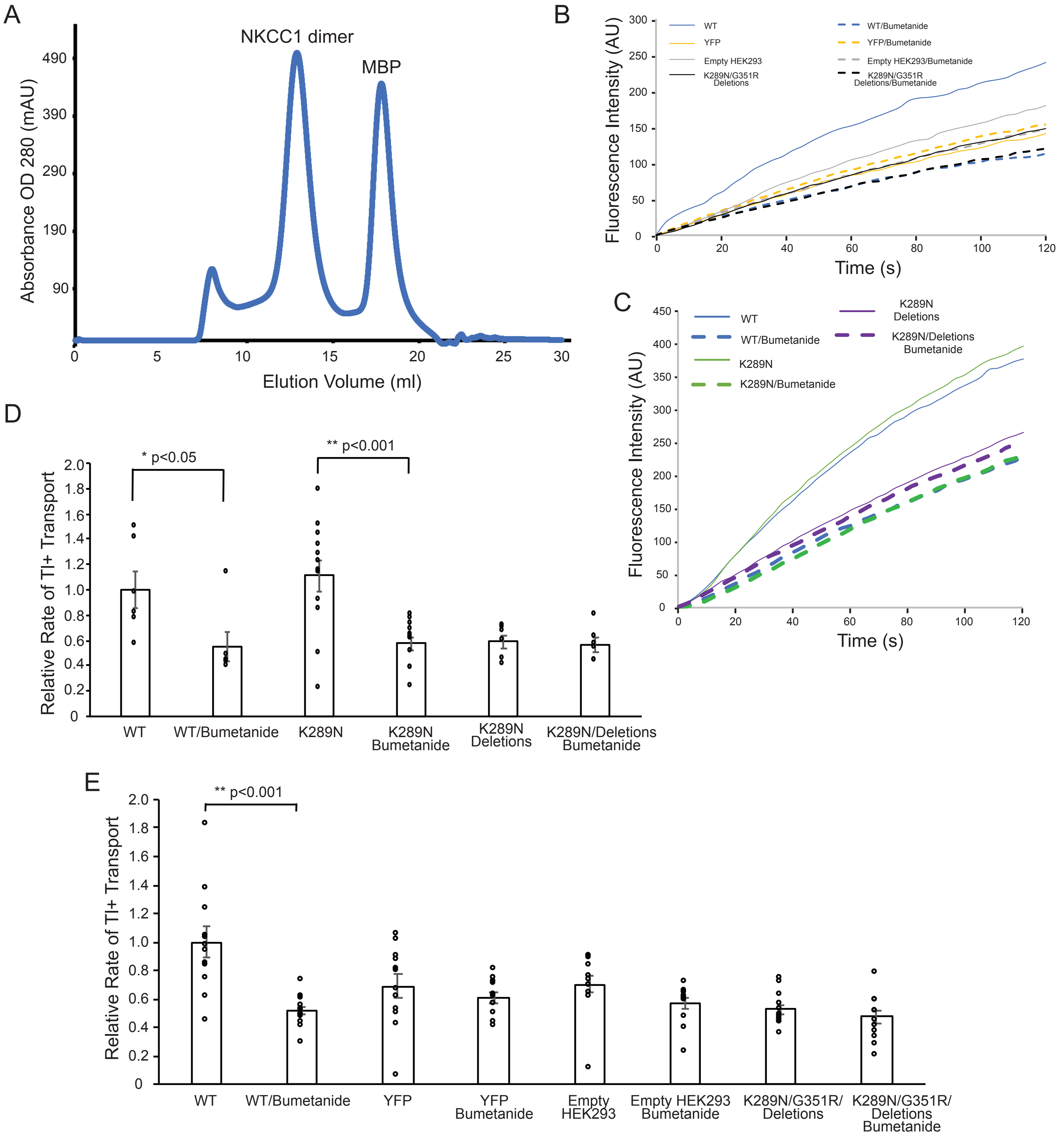

Supplementary Figure 1 | Expression and functional characterization of NKCC1.

(A) Size exclusion chromatogram of the optimized human NKCC1 construct (K289N/G351R/deletions). The peaks corresponding to the transporter or maltose binding protein tag are labeled.

(B-C) Kinetics of transport were measured for the WT NKCC1, YFP-expressing or empty HEK293 control, and three NKCC1 mutants using Tl^{+} ion flux assay.

(D-E) Rates of transport were all normalized to that measured for the WT NKCC1 without inhibition by bumetanide. All human NKCC1 variants, except for the K289N mutant, as well as two controls (i.e., YFP-expressing or empty HEK293 cells) show no statistically significant bumetanide-sensitive transport. Each circle represents a measurement of an independent experiment and error bars represent s.e.m.

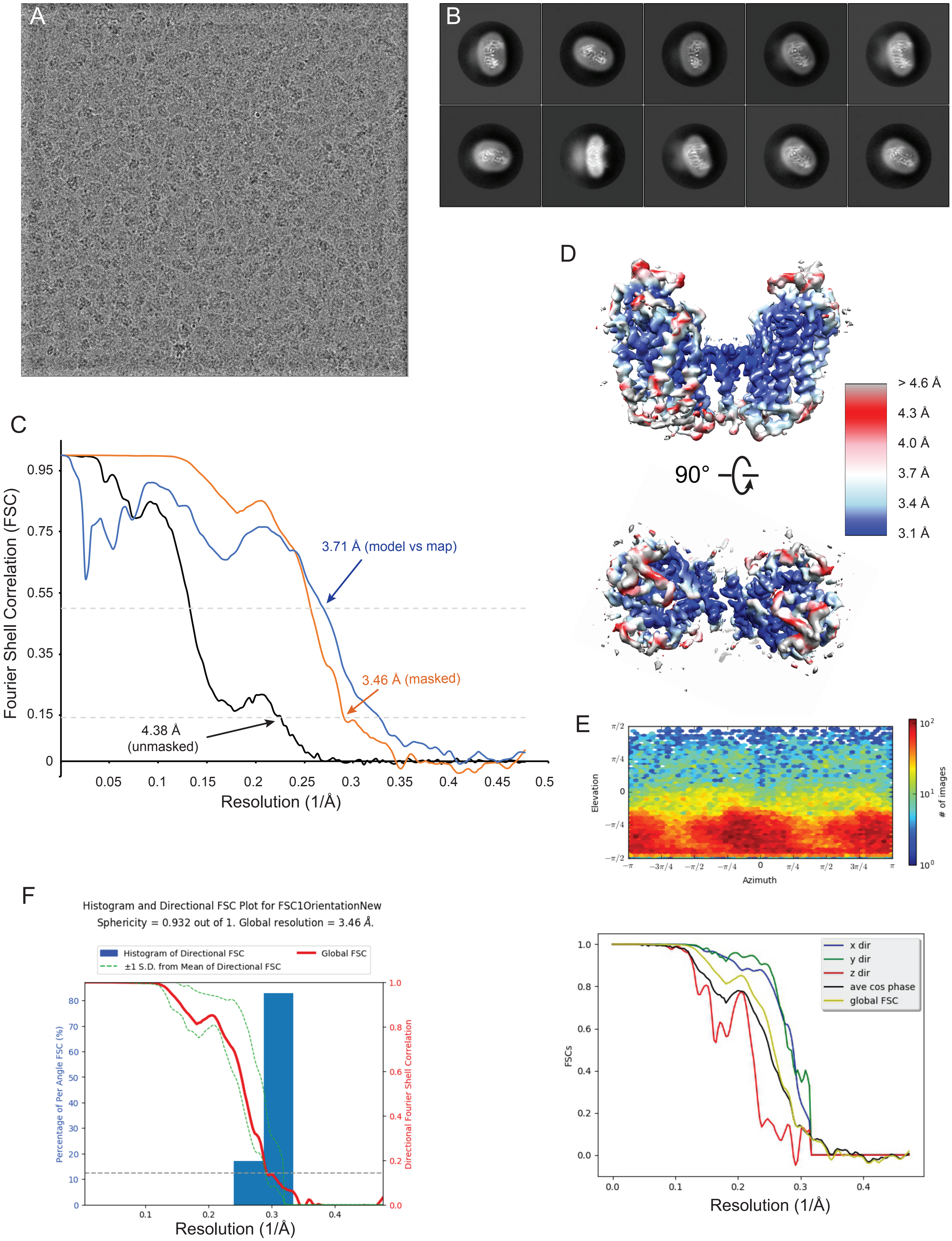
A
B
C
Fourier Shell Correlation (FSC)
Resolution (1/Å)
3.71 Å (model vs map)
3.46 Å (masked)
4.38 Å
(unmasked)
D
90°
> 4.6 Å
4.3 Å
4.0 Å
3.7 Å
3.4 Å
3.1 Å
E
Elevation
Azimuth
# of images
F
Histogram and Directional FSC Plot for FSC1OrientationNew
Sphericity = 0.932 out of 1. Global resolution = 3.46 Å.
Histogram of Directional FSC
±1 S.D. from Mean of Directional FSC
Global FSC
Percentage of Per Angle FSC (%)
Directional Fourier Shell Correlation
Resolution (1/Å)
x dir
y dir
z dir
ave cos phase
global FSC
FSCs
Resolution (1/Å)

Supplementary Figure 2 | Human NKCC1 structure determined by single particle cryo-EM.

(A) A representative micrograph of human NKCC1 recorded with a Krios microscope.

(B) 2D classes of human NKCC1 show well resolved structural features for the transmembrane domain, but only fuzzy densities for the cytosolic domains.

(C) Gold-standard FSC curves calculated after cryosparc non-uniform auto-refinement.

(D) Local resolution calculated in cryosparc.

(E) Angular distribution plot of all particle projections as output by cryosparc.

(F) Directional resolutions were calculated using a remote 3DFSC processing server.

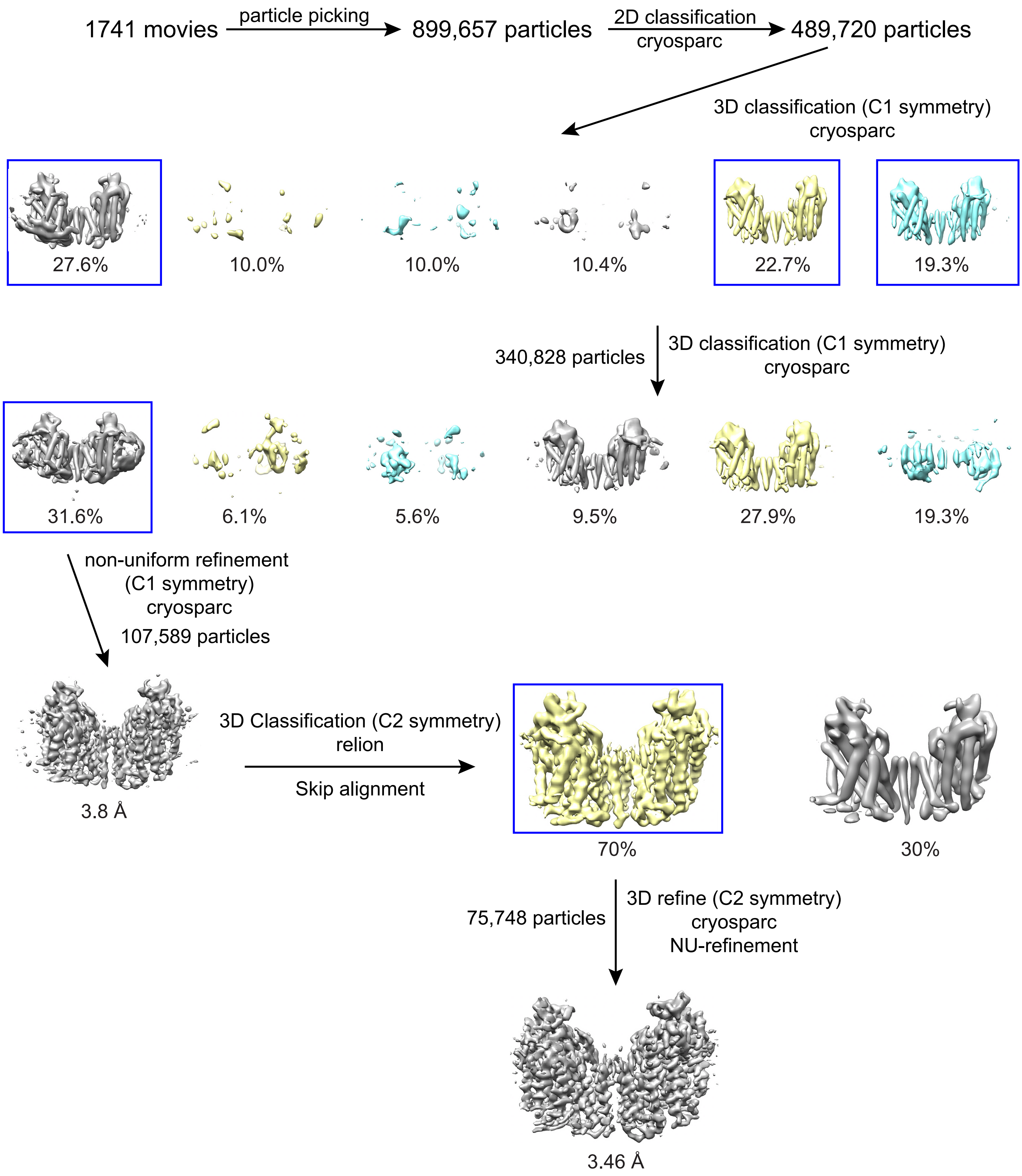

Supplementary Figure 3 | 3D reconstruction of human NKCC1.
Flow chart of image processing for NKCC1. Note that both RELION and cryosparc were used for reconstruction.

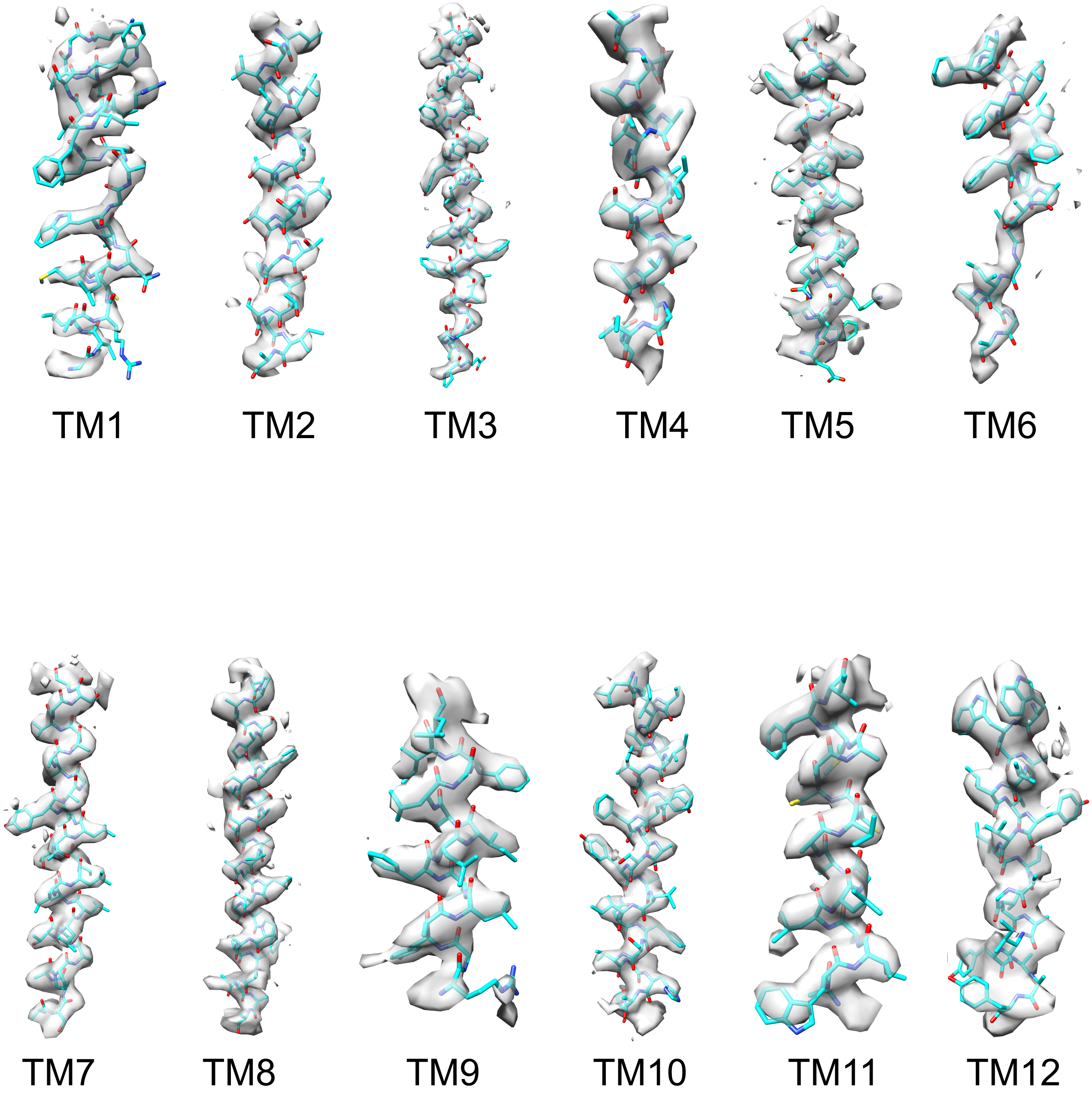

Supplementary Figure 4 | EM density maps of transmembrane helices of the human NKCC1 transporter. The map is locally sharpened with an overall B factor of -100 Å2 in cryosparc. The final model shown in sticks is docked into experimental densities.

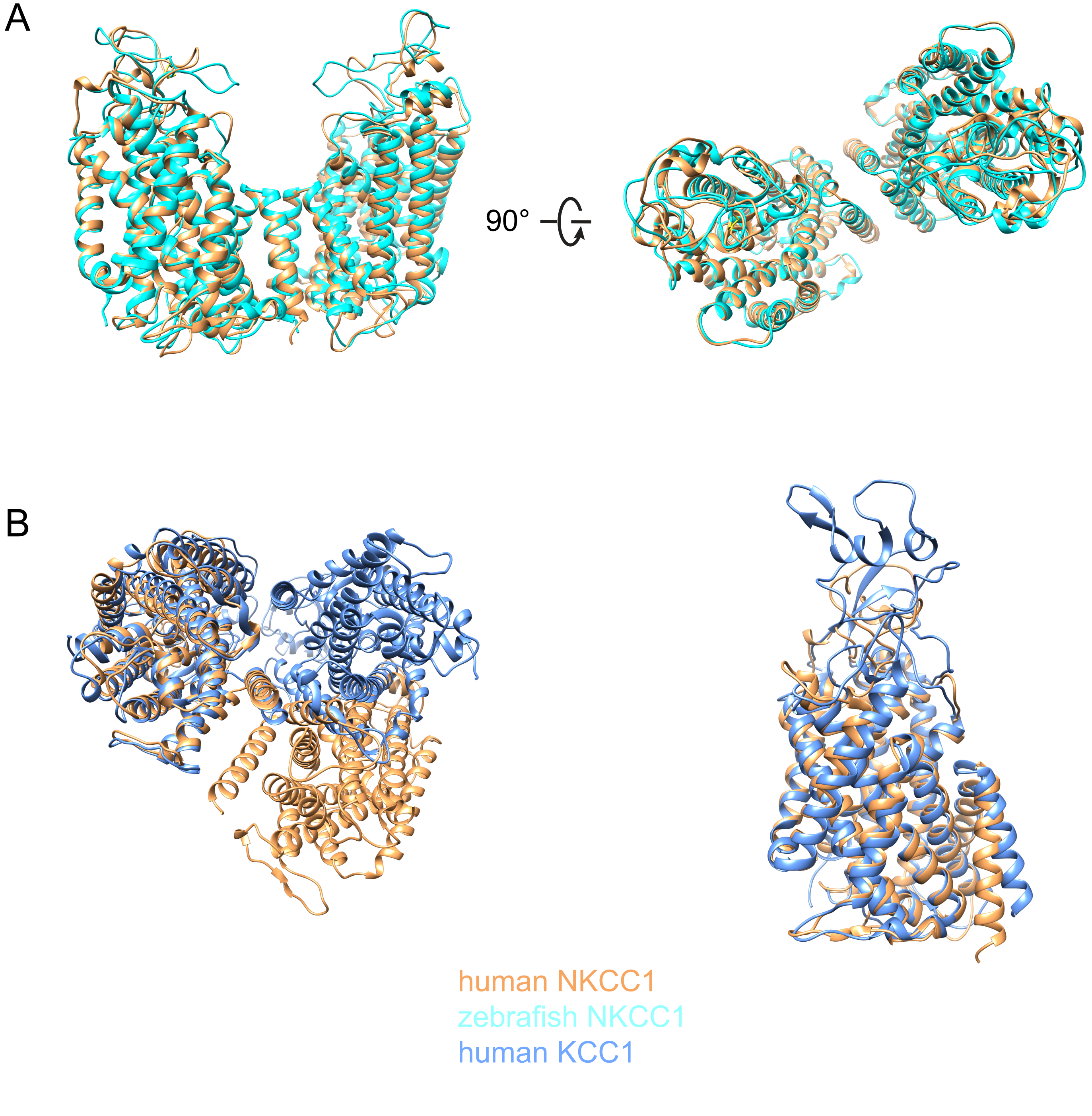

Supplementary Figure 5 | Comparison of human and zebrafish NKCC1 structures.
(A) Side (left) and extracellular (right) views show that human and zebrafish NKCC1 structures largely superimpose within their transmembrane regions, but exhibit notable differences within the extracellular domains and loops connecting transmembrane helices.
(B) Human NKCC1 and KCC1 adopt a drastically different dimeric architecture (left), although a single subunit of these transporters superimposes nicely within the transmembrane region (right).

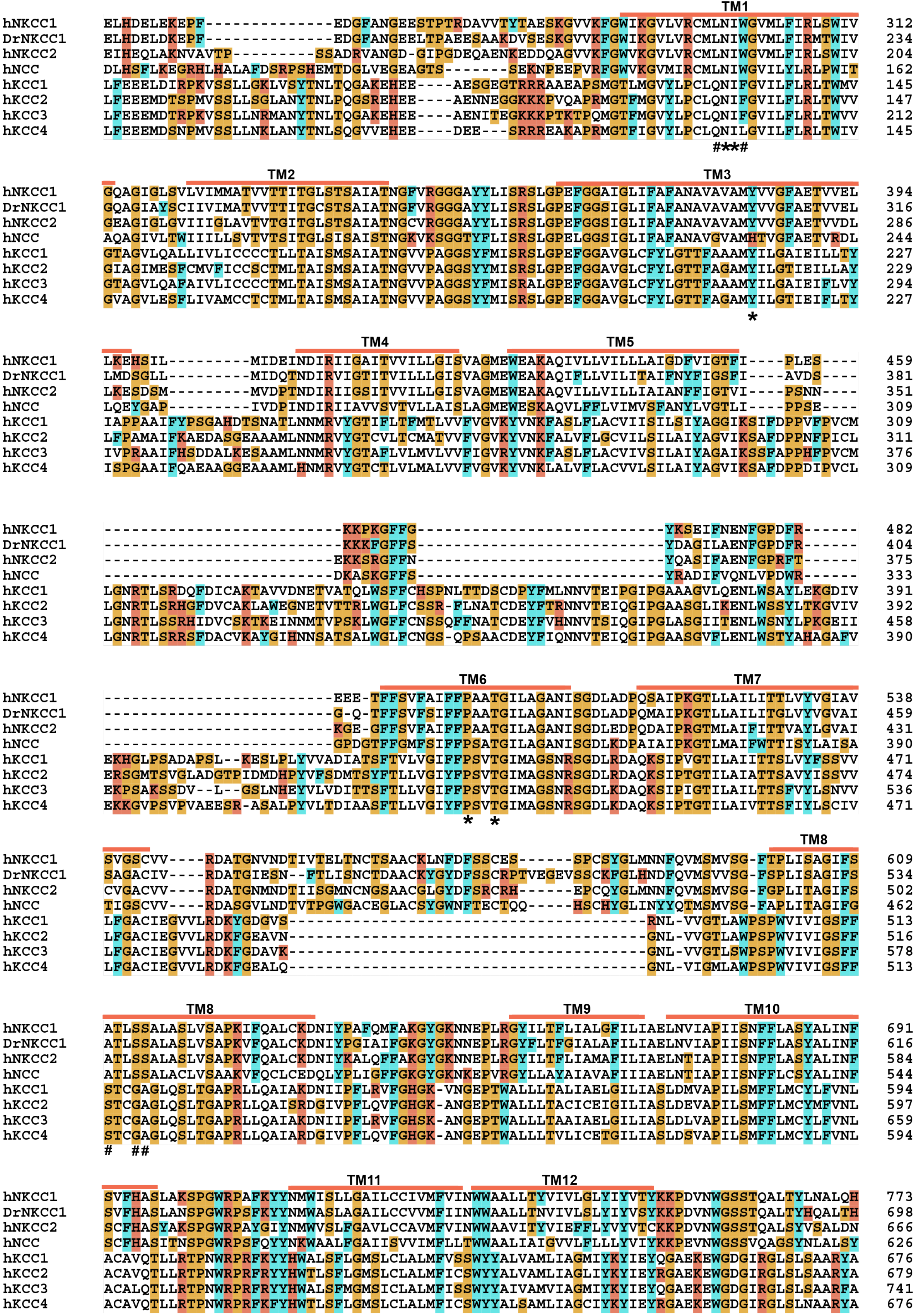

Supplementary Figure 6 | Alignment of sequences of CCC transporters. Transmembrane helices are denoted with red lines. Na^+ and K^+ binding sites are highlighted with # and *, respectively.

Supplementary Table 1 | Primers used in this study

hNKCC1 YFP Forward primer:
TCAAGGTCCAGGATCCGAGCCGAGGCCCACGGCTCCT

hNKCC1 NotI Reverse primer:
GATTCGAAAGCGGCCGCTTATGAATAGAAGGTAAGGACACT

hNKCC1 G351R Forward primer: GGAGGCCGCGCATATTATTTAATATCTAGA

hNKCC1 G351R Reverse primer: ATATGCGCGGCCTCCCCTTACAAATCCATT

hNKCC1 K289N Forward primer: GGATCAACGGTGTACTAGTACGTTGTATG

hNKCC1 K289N Reverse primer: GTACACCGTTGATCCAGCCAAACTTCACGAC

hNKCC1 R294A Forward primer: CTAGTAGCTTGTATGTTAAACATTTGGGGT

hNKCC1 R294A Reverse primer: CATACAAGCTACTAGTACACCCTTGATCCA

hNKCC1 Δ (254-278) Forward primer: AAAGGAAAGTAAAGGAGTCGTGAAGTTTGGC

hNKCC1 Δ (254-278) Reverse primer: CCTTTACTTTCCTTTTCCAGCTCGTCATGCAG

hNKCC1 Δ (941-1000) Forward primer: TGTCATCATTGAATGTAGCTGACCAAAAG

hNKCC1 Δ (941-1000) Reverse primer: CATTCAATGATGACAATAATTCTTCTTG

Supplementary Table 2 | Statistics of cryo-EM and model building

**Data collection/Processing**	
Voltage (kV)	300
Magnification	47,619
Defocus range (μm)	-1.0 – -3.5
Pixel size (Å)	1.05
Total electron dose (e^-/Å^2)	56
Exposure time (s)	9
Number of images	1,741
Number of frames per image	36
Initial particle number	899,657
Final particle number	75,748
Resolution (unmasked, Å)	4.38
Resolution (masked, Å)	3.46
**Refinement and Validation**	
Number of atoms	6,062
R.M.S deviation	
Bond length (Å)	0.006
Bond angles (°)	0.701
Ramachandran	
Favored (%)	88.15%
Allowed (%)	11.42%
Outlier (%)	0.43%
Molprobity score	2.20
