# Supplementary figures and images for: Structure of the human cation–chloride cotransporter NKCC1 determined by single-particle electron cryo-microscopy
Source: Nat Commun. 2020 Feb 21;11:1016. doi: 10.1038/s41467-020-14790-3 (PMC7035313; doi:10.1038/s41467-020-14790-3)

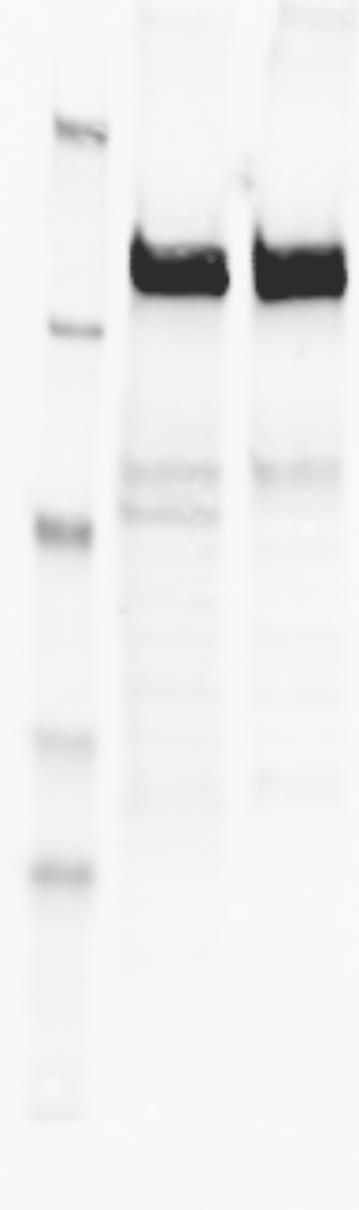

Supplement: Supplementary file 3 — Source Data [file 41467_2020_14790_MOESM3_ESM.zip › 221055_3_data_set_4373619_q4m8b9.tif]
